# Tracking Monocyte Recruitment and Macrophage Accumulation in Atherosclerotic Plaque Progression Using a Novel hCD68GFP/ApoE^−/−^ Reporter Mouse—Brief Report

**DOI:** 10.1161/ATVBAHA.116.308367

**Published:** 2017-01-25

**Authors:** Eileen McNeill, Asif J. Iqbal, Daniel Jones, Jyoti Patel, Patricia Coutinho, Lewis Taylor, David R. Greaves, Keith M. Channon

**Affiliations:** From the Division of Cardiovascular Medicine, British Heart Foundation Centre for Research Excellence, Radcliffe Department of Medicine, John Radcliffe Hospital, United Kingdom (E.M., D.J., J.P., P.C., K.M.C.); Wellcome Trust Centre for Human Genetics, Headington, Oxford, United Kingdom (E.M., J.P., P.C., K.M.C.); and Sir William Dunn School of Pathology, University of Oxford, United Kingdom (A.J.I., L.T., D.R.G.).

**Keywords:** atherosclerosis, GFP, macrophage, model, monocyte, mouse, trafficking

## Abstract

**Objective—:**

To create a model of atherosclerosis using green fluorescent protein (GFP)–targeted monocytes/macrophages, allowing analysis of both endogenous GFP^+^ and adoptively transferred GFP^+^ myeloid cells in arterial inflammation.

**Approach and Results—:**

hCD68GFP reporter mice were crossed with ApoE^−/−^ mice. Expression of GFP was localized to macrophages in atherosclerotic plaques and in angiotensin II–induced aortic aneurysms and correlated with galectin 3 and mCD68 expression. Flow cytometry confirmed GFP^+^ expression in CD11b^+^/CD64^+^, CD11c^+^/MHC-II^HI^, and CD11b^+^/F4/80^+^ myeloid cells. Adoptive transfer of GFP^+^ monocytes demonstrated monocyte recruitment to both adventitia and atherosclerotic plaque, throughout the aortic root, within 72 hours. We demonstrated the biological utility of hCD68GFP monocytes by comparing the recruitment of wild-type and CCR2^−/−^ monocytes to sites of inflammation.

**Conclusions—:**

hCD68GFP/ApoE^−/−^ mice provide a new approach to study macrophage accumulation in atherosclerotic plaque progression and to identify cells recruited from adoptively transferred monocytes.

## Abstract 2

Supplemental Digital Content is available in the text.

Monocyte/macrophage biology is a key driver of atherosclerotic disease.^1^ Recent observations highlight the importance of the origin and proliferation of macrophages within diseased arteries, and alterations in macrophage biology mediate plaque regression.^2–4^ The recently described hCD68GFP reporter mouse has abundant and persistent green fluorescent protein (GFP) expression in both circulating monocytes and tissue macrophages.^5^ Because atherosclerosis is a complex vascular disease, with disease development occurring in multiple locations and analysis being heavily reliant on imaging or fluorescence technologies (histology, microscopy, and flow cytometry), we hypothesized that high-level GFP expression within monocyte/macrophage populations would make the hCD68GFP mouse a powerful tool for probing myeloid cell biology in atherosclerotic plaque progression.

## Materials and Methods

We used hCD68GFP mice,^5^ which we have made available via Jackson Laboratories (Bar Harbor, ME), and ApoE^−/−^ mice (Charles River UK Ltd) on C57Bl/6 background. Details of the methods can be found in the online-only Data Supplement.

## Results

**Figure 1.**
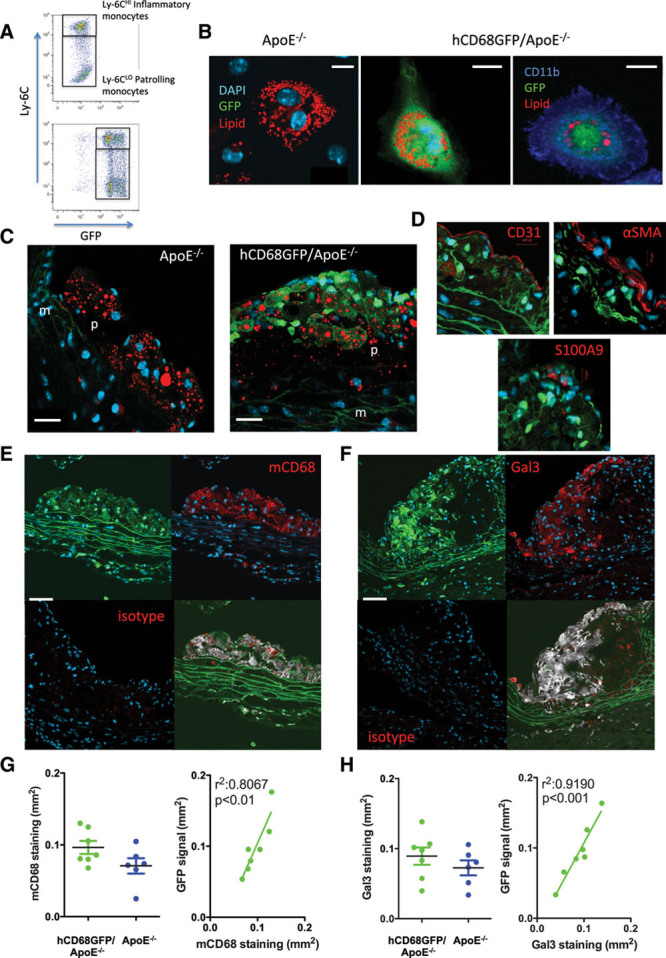
hCD68GFP/ApoE^−/−^ mice show abundant green fluorescent protein (GFP) expression in arterial lesions. **A**, Blood samples were harvested and stained to identify CD45^+^/CD115^+^/Ly6G^-^ monocytes of both the Ly6C^HI^ and Ly6C^LO^ phenotype. Compared with ApoE^−/−^ mice, the hCD68GFP/ApoE^−/−^ mice have a clear increase in GFP fluorescence in both monocyte populations. **B**, Two percent thioglycollate solution was intraperitoneally injected into mice that had been on a high-fat diet for at least 8 weeks. Four days after injection, the elicited peritoneal cells were harvested and macrophages selected by adhesion to glass coverslips. Cells were fixed and stained with Lipidtox Red (red) to identify neutral lipid droplets and DAPI to visualize nuclei (blue), with cells being confirmed as macrophages by cell surface staining for CD11b expression (**Right** panel, blue) (scale bar, 10 μm). **C**, The aortic root from mice that had been maintained on a high-fat diet for 10 weeks was fixed with 4% formaldehyde and cut into 7 μm frozen sections. Staining for neutral lipids (red) revealed areas of strong GFP fluorescence (green) localized to lipid containing plaques only from mice harboring the hCD68GFP transgene, compared to ApoE^−/−^ controls (p, plaque; m, media; DAPI, blue; scale bar, 20 μm). **D**, Bright GFP fluorescence did not colocalize with markers of endothelial cells (CD31), infiltrating smooth muscle cells (α-smooth muscle actin), or neutrophils (S100A9). **E** and **F**, Strong colocalization of GFP (green) at the cellular level was seen when sections were costained with anti-macrophage antibodies (targeted against mCD68 or Galectin 3; red) and DAPI (blue; scale bar, 20 μm). A lack of staining in isotype control sections is shown in the lower left panel. Colocalization is shown (gray) in the lower **right** panel, overlap with DAPI was removed to prevent any spillover, then pixels were assigned as green or red or neither using a threshold set on the non-GFP expressing/unstained controls. Pixels that were assigned both green and red were colored gray in the resulting image. **G** and **H**, Quantification of the area of mCD68 and Gal3 staining within plaque from hCD68GFP/ApoE^−/−^ and ApoE^−/−^ mice showed no significant effect of GFP expression on macrophage infiltration into plaque and a highly significant correlation between GFP and mCD68/Gal3 staining. n=6 to 7, Students *t* test; *P*<0.05 regarded significant. Linear regression analysis detected a highly significant relationship between GFP and mCD68/Gal3; *P*<0.05.

We crossed hCD68GFP mice with ApoE^−/−^ mice to produce a line of homozygous ApoE^−/−^ mice carrying a single copy of the hCD68-GFP locus. Expression of GFP was present in both Ly-6C^HI^ and Ly-6C^LO^ blood monocytes, as is the case in normolipidemic mice (Figure 1A). Because GFP requires an aqueous environment for fluorescence, we confirmed that a clear signal could also be seen in lipid-laden foam cells (Figure 1B). Exclusion of GFP from the lipid droplets was clear, but the remaining cytosol provided a bright fluorescent transgene signal. Lipid-laden cells were confirmed as macrophage-derived foam cells by coexpression of CD11b. We next examined the fluorescence of cells within aortic root plaque from animals fed a high-fat diet for 10 weeks. GFP fluorescence was clearly visible within aortic root lesions, associated with areas of lipid accumulation (Figure 1C). This signal colocalized at a cellular level with the macrophage markers mCD68 and galectin 3 (mac2 epitope), but not other plaque components (Figure 1D through 1F), using a thresholded colocalization analysis. Analysis of the colocalization in the luminal plaque showed a Mander’s overlap coefficient of M1: 0.923 and M2: 0.978, indicating a good coincidence of the green and red channel over the total intensity. Of note, at a cellular level, cells with the highest GFP expression did not necessarily express the highest levels of the macrophage markers, indicating that GFP expression is heterogeneous within the myeloid populations within the plaque. No colocalization with GFP fluorescence was seen with CD31, S100A9, or alpha smooth muscle actin to identify endothelial cells, neutrophils, or smooth muscle cells within plaque (Figure 1D), although this does not exclude a low-level fluorescence in other cell populations, as has been previously reported in neutrophils.^5^

We next performed quantitative analysis of the GFP signal versus mCD68 and Gal3 staining using fluorescence microscopy. We observed a strong correlation between the GFP fluorescence and macrophage markers; plaque macrophage content was not significantly affected by the hCD68GFP transgene (Figure 1G and 1H). Additionally, there was no significant difference in macrophage infiltration in the aortic root plaques between GFP-expressing and nonexpressing littermates or in total plaque area, as measured using Masson’s Trichrome stain (Figure 1G and 1H; Figure IA and IB in the online-only Data Supplement). A similar correlation between macrophage content and GFP fluorescence, with a lack of effect on total or macrophage plaque area, was also observed in smaller plaques found in ApoE^−/−^ animals fed a chow diet for 16 weeks (Figure IA through IC in the online-only Data Supplement). There was no confounding effect of hCD68-GFP on plasma lipid levels (high-fat diet: total cholesterol, 41.2 mmol/L [±2.01; GFP^+^] versus 40.3 mmol/L [±3.44; GFP^−^]; high-density lipoprotein, 2.4 mmol/L [±0.09; GFP^+^] versus 2.4 mmol/L [±0.08; GFP^−^]; Chow: total cholesterol, 6.9 mmol/L [±1.3; GFP^+^] versus 6.5 mmol/LL [±0.8; GFP^−^]; high-density lipoprotein, 1.27 mmol/L [±0.1; GFP^+^] versus 1.33 mmol/L [±0.3; GFP^−^]).

The brightness of GFP expression in monocyte/macrophages from the hCD68GFP/ApoE^−/−^ mouse enabled whole-mount en-face analysis of the aorta, revealing plaque macrophages above the autofluorescence of the elastic lamina (Figure IIA through IIC in the online-only Data Supplement). To identify these GFP^+^ foam cells, en-face preparations were permeabilized and stained with a mCD68 antibody, demonstrating that the large GFP^+^ cells present were macrophages (Figure IIA in the online-only Data Supplement). A stacked image revealed typical orientation of the CD31^+^ endothelial cell layer in the nonatherosclerosis-prone outer curvature of the aortic arch (Figure IIB in the online-only Data Supplement). In contrast, imaging the inner curvature revealed a highly disorganized endothelium, with large clusters of GFP^+^ foam cells. We reconstructed images of the entire vessel wall, revealing that the GFP fluorescence was sufficiently bright to detect the accumulation of adventitial macrophages, adjacent to sites of arterial inflammation (Figure IIC and Movie file in the online-only Data Supplement). The brightness of the transgene GFP signal will enable new studies of the cross talk between luminal and adventitial inflammation in atherogenesis.

The infusion of angiotensin II into hCD68GFP/ApoE^−/−^ mice caused a rapid recruitment of GFP^+^ myeloid cells to the aortic wall within 5 days (Figure III in the online-only Data Supplement). Prolonged infusion of angiotensin II caused aneurysm formation, with abundant GFP^+^ cells in the vessel adventitia and hematoma border that coexpress mCD68 (Figure III in the online-only Data Supplement).

**Figure 2.**
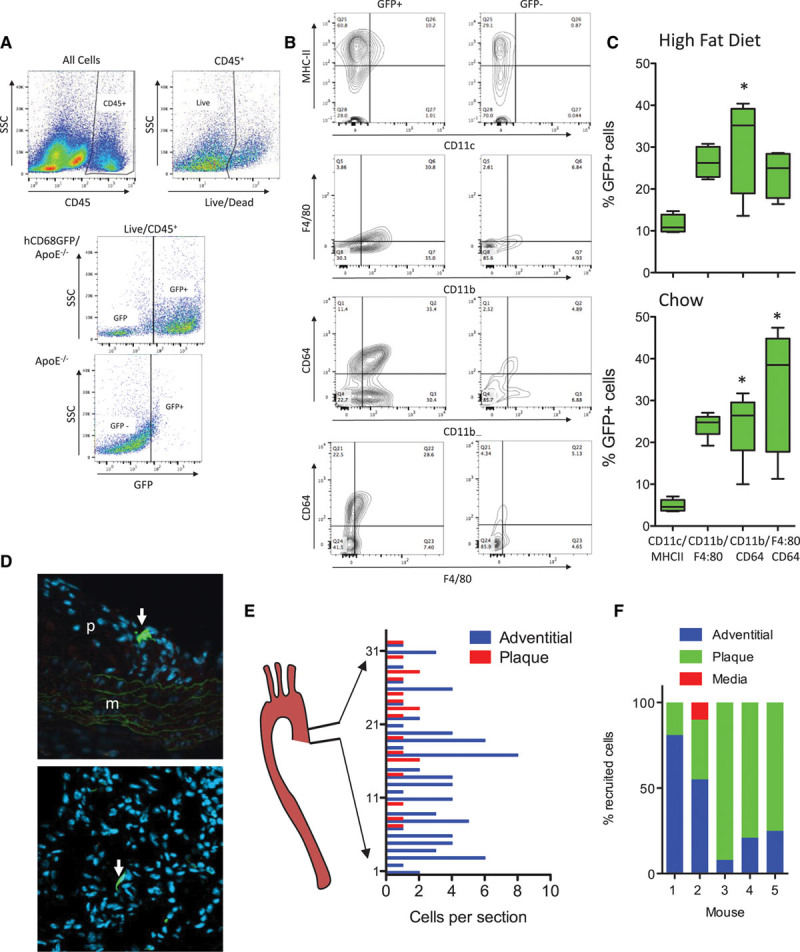
hCD68GFP/ApoE^−/−^ mice enable identification of multiple myeloid populations in aortic lesions and allow tracking of adoptively transferred monocytes. Aortic digests demonstrate the presence of green fluorescent protein (GFP) expression in multiple myeloid populations within the descending aorta from mice fed a high-fat diet for 10 weeks and harvested at 24 weeks of age (female). Aortas were digested using a standard digest mixture (collagenase I, collagenase XI, hyaluronidase, and DNAse I), with the resulting single cell suspension being stained with a viability dye and antibody cocktail to identify macrophage/dendritic cell populations (CD45, CD11b, CD64, CD11c, MHC-II, F4/80) by comparison to isotype control samples or GFP^−^ controls. **A**, Viable leukocytes were identified as CD45+/live cells, and GFP^+^ cells were gated by comparison to GFP^−^ samples. **B**, Both GFP^−^ and GFP^+^ populations within the Live/CD45 population were gated to identify the presence of myeloid cell populations within these 2 populations. The MHC-II/CD11c, CD11b/F4/80, and CD64/CD11b populations were found primarily within the GFP^+^ population. **C**, The relative contribution of the 3 populations to the total GFP^+^ population in aortas from both high fat–fed and chow-fed (16-week female mice; see Figure IV in the online-only Data Supplement) mice were quantified (1-way ANOVA with Dunn’s Multiple Comparison post test; *P<0.05 vs CD11c/MHC-II group, all other pairwise comparisons not significantly different; box and whisker plot max–min, n=4–5). **D**, 1.5×10^6^ GFP^+^ monocytes isolated from bone marrow by negative selection were injected intravenously into mice that had been maintained on a high-fat diet for 8 weeks. Tissue samples were harvested 72 h later and the aortic root cut into frozen sections and stained with DAPI (blue) and GFP^+^ cells (green) visualized and counted in every second slide (GFP^+^ cells highlighted by white arrows). **E**, The number of GFP^+^ cells present per complete section analyzed throughout the aortic root from heart-aorta was plotted. **F**, Representative data from 5 adoptive transfer recipients demonstrated recruitment to both plaque and adventitial sites.

While analysis of tissue sections is a mainstay of atherosclerotic analysis, detailed phenotyping of the cell populations in atherosclerotic plaques requires flow cytometry after tissue digests. We used a panel of antibodies that differentiate key myeloid cell populations to characterize in more detail the GFP^+^ cell types within the vessel wall. Interrogation of the live CD45^+^ population revealed that typically 54% (chow) to 60% (high fat fed) of the total leukocyte population in ApoE^−/−^ aortae are GFP^+^ (Figure 2A and data not shown). When the GFP^+^ or GFP^−^ CD45^+^ cells present were analyzed within each hCD68GFP-ApoE^−^^/−^ animal, it was clear that the majority of macrophage populations were present within the GFP^+^ population, including those expressing markers CD11b^+^/CD64^+^ and the CD11b^+^/F4/80^+^ and CD11c^+^/MHC-II^HI^ subpopulations (Figure 2A through 2C). These GFP^+^ cell populations were found in both chow-fed and high-fat diet–fed animals (Figure 2A and 2B; Figure IV in the online-only Data Supplement). Monocytes present within the aorta were also GFP^+^, as shown in aortic digests from angiotensin II–infused mice (Figure IV in the online-only Data Supplement).

A major application of the hCD68GFP reporter mouse is the ability to track cells after adoptive transfer.^5^ Given the high signal-to-background GFP fluorescence, we reasoned that adoptive transfer of monocytes into ApoE^−^^/−^ mice would allow robust identification of individual monocytes migrating into atherosclerotic plaque. We performed monocyte isolation by monocyte enrichment using no touch antibody depletion of other cell types from blood, bone marrow, and spleen, selecting bone marrow as the source yielding the greatest number and purity of monocytes (Figure V in the online-only Data Supplement). Adoptive transfer of bone marrow monocytes, into high fat–fed animals with established disease, was performed 72 hours prior to harvest. Analysis of sections throughout the entire aortic root revealed that GFP^+^ cells could be clearly visualized within the aortic root (Figure 2D). Adoptively transferred cells were observed both in the adventitia, where the cells had an elongated morphology with minimal protrusions, and within plaques, where cells had a spread morphology with multiple protrusions. Systematic quantification of the location of adoptively transferred monocytes revealed recruited cells in both the adventitia and the plaque throughout the aortic root, with recruitment to plaque being more prominent in the upper portion of the root (Figure 2E). The relative predominance of recruitment to plaque or adventitia varied between animals, indicating that this model may be useful in determining the factors that control and influence sites of monocyte recruitment using laser capture microscopy or recovery of GFP^+^ cells by fluorescence-activated cell sorting (Figure 2F).

In addition to allowing the study of these rare cells, we also tested the utility of adoptive monocyte transfer into acute inflammation models, such as sterile peritonitis, with quantification of genotype-specific effects on the donor cells. Although CCR2^−/−^ mice have significantly reduced proportions of both Ly6C^HI^ and Ly6C^LO^ monocytes,^6^ isolation of bone marrow yielded cell samples with similar proportions of Ly6C^HI^ monocytes (30% versus 25.5%), enabling us to directly compare recruitment of these cells into wild-type CCR2 replete host animals (Figure VI in the online-only Data Supplement). We compared transfer of hCD68GFP versus hCD68GFP/CCR2^−^^/−^ monocytes into wild-type recipient animals with zymosan-induced peritonitis. We showed that recruitment of CCR2^−^^/−^ monocytes was strikingly reduced, by 75%, compared with that of CCR2^+/+^ monocytes (Figure VI in the online-only Data Supplement).

## Discussion

We report a novel hCD68GFP/ApoE^−^^/−^ mouse model to quantify macrophage accumulation within atherosclerotic plaques and to track hCD68GFP^+^ monocyte recruitment in adoptive transfer studies. High-level monocyte GFP expression, that is sustained on differentiation into macrophages and foam cells, underpins the ability to perform long-term adoptive transfer and plaque progression/regression studies with this transgenic reporter mouse.^5^ This is in contrast to other myeloid cell reporter lines such as CX_3_CR1-GFP, which we have previously shown loses GFP fluorescence during macrophage differentiation, and the CD11cYFP mice, which show YFP (yellow fluorescent protein) transgene expression that is limited to splenic dendritic cells and alveolar macrophages (Figure VII in the online-only Data Supplement).^5,7^ In contrast, the hCD68GFP mouse shows robust GFP expression in monocytes, macrophages, and dendritic cells (Figure VII in the online-only Data Supplement). Analysis of in vitro bone marrow–derived macrophages and dendritic cells demonstrated that both cell types expressed GFP, with expression of YFP in the CD11cYFP cells providing a much less intense signal in both cell types (Figure VIII in the online-only Data Supplement).

We demonstrate that detection of even rare cells in the artery wall is feasible, given the brightness of GFP expression, even in complex vascular pathologies. The use of either radioactive labeling, bone marrow chimerism, parabiosis, or the loading of monocytes with beads (after systemic clodronate liposome administration) have been the primary methods used to directly assess the kinetics of monocyte recruitment to plaque.^8,9^ However, these methods all have limitations of sensitivity or a requirement for a systemic treatment that may profoundly alter the inflammatory biology of the plaque—such as depletion of endogenous cells or major surgery. In contrast, we can conclusively demonstrate that the GFP^+^ cells found in plaque were recruited within a 72-hour window showing that monocytes have ongoing and relatively rapid access to the disease tissue. The genetic marking of these cells, rather than bead labeling that can be transferred between cells, will allow studies of the life time and turnover of these cells. Furthermore, the unique advantages of hCD68GFP/ApoE^−^^/−^ mice will enable studies of single cells for gene expression analysis, without multiple layers of antibody staining prolonging the time between cell isolation and RNA extraction.

## Acknowledgments

CD11cYFP mice were kindly provided by Dr A. Spencer, The Jenner Institute, University of Oxford. Information on the hCD68-GFP mouse can be found at http://www.cardioscience.ox.ac.uk/hcd68-gfp-transgenic-mouse.

## Sources of Funding

This work was supported by the British Heart Foundation (RG/15/10/31485), (RG/12/5/29576), and (FS/12/69/30008), Wellcome Trust (090532/Z/09/Z), and the National Institute for Health Research (NIHR) Oxford Biomedical Research Centre. K.M. Channon and E. McNeill acknowledge support from the BHF Centre of Research Excellence, Oxford (RE/13/1/30181), in supporting Mr Daniel Jones via a summer research studentship.

## Disclosures

None.

## Supplementary Material

## Highlights

- The hCD68GFP reporter mouse, crossed with ApoE^−/−^ mice, enables new studies of monocyte recruitment and macrophage accumulation, processes that are critical drivers of atherosclerotic disease.
- The high level of green fluorescent protein fluorescence in circulating monocytes, macrophages, and foam cells is sufficient to allow clear discrimination above background in both tissue sections and whole-mount en-face aortic preparations, despite the known autofluorescence of these tissues.
- hCD68GFP^+^ monocytes adoptively transferred into mice with atherosclerosis are recruited to both plaque and adjacent adventitia within 72 hours of injection.
