# Supplementary material for: Tracking Monocyte Recruitment and Macrophage Accumulation in Atherosclerotic Plaque Progression Using a Novel hCD68GFP/ApoE−/− Reporter Mouse—Brief Report
Source: Arterioscler Thromb Vasc Biol. 2017 Jan 25;37(2):258–63. doi: 10.1161/ATVBAHA.116.308367 (PMC5274540; doi:10.1161/ATVBAHA.116.308367)
Supplement: Supplementary file 1 [file atv-37-258-s001.pdf]

Data Supplement

Tracking monocyte recruitment and macrophage accumulation in atherosclerotic plaque progression using a novel hCD68-GFP/ApoE$^{-/-}$ reporter mouse

Eileen McNeill[1,2*], Asif J. Iqbal[3*], Daniel Jones[1], Jyoti Patel[1,2], Patricia Coutinho[1,2], Lewis Taylor[3], David R. Greaves[3], Keith M. Channon[1,2]

[1] Division of Cardiovascular Medicine, British Heart Foundation Centre for Research Excellence, John Radcliffe Hospital,
[2] Wellcome Trust Centre for Human Genetics, and
[3] Sir William Dunn School of Pathology, University of Oxford
* These authors contributed equally to this manuscript.

Corresponding author: Dr Eileen McNeill, Division of Cardiovascular Medicine, British Heart Foundation Centre for Research Excellence, John Radcliffe Hospital, University of Oxford, Oxford, OX3 9DU, U.K.

Tel: +44 1865 287662 Email: eileen.mcneill@well.ox.ac.uk

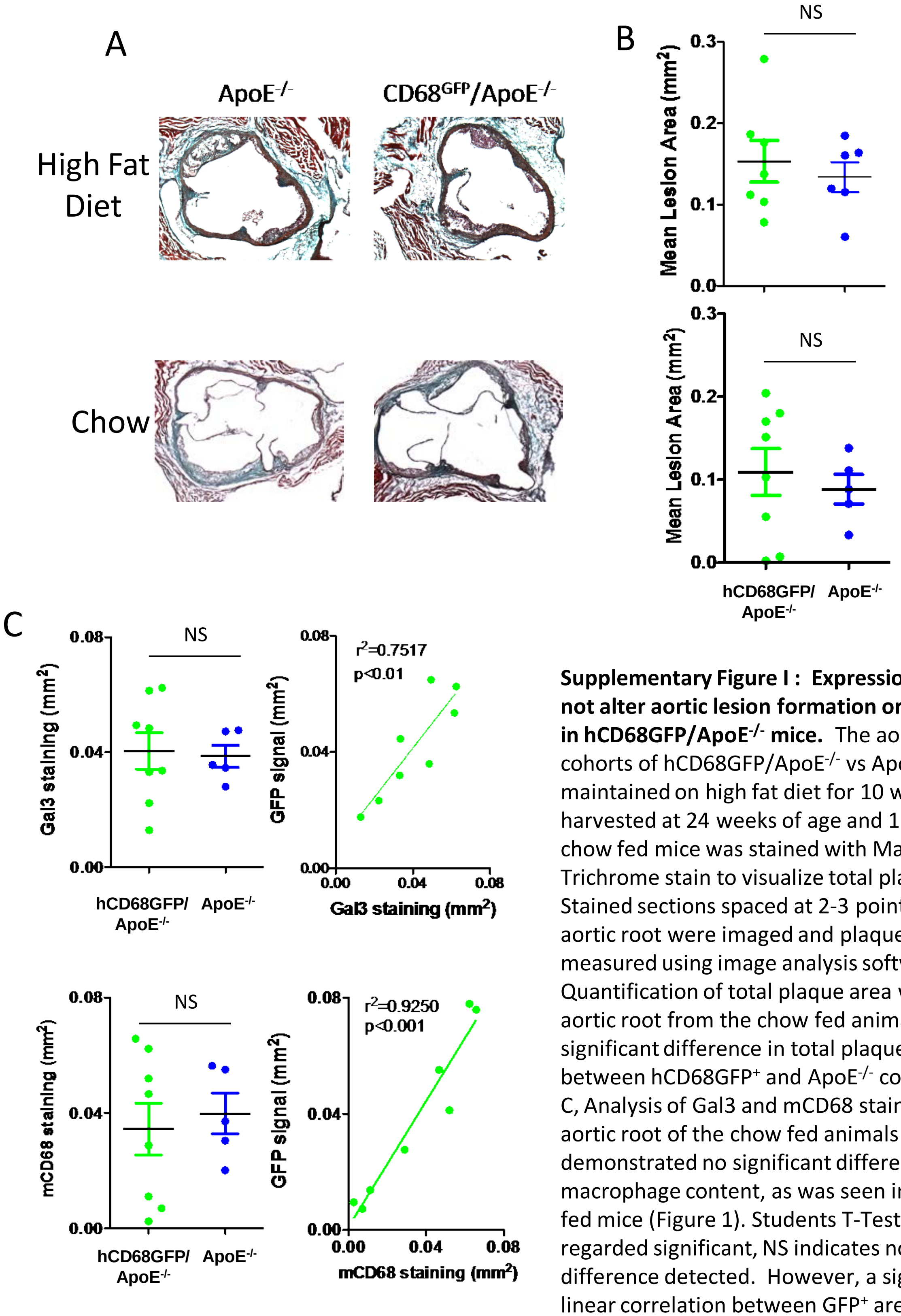

Supplementary Figure I : Expression of GFP does not alter aortic lesion formation or composition in hCD68GFP/ApoE$^{-/-}$ mice. The aortic root from cohorts of hCD68GFP/ApoE$^{-/-}$ vs ApoE$^{-/-}$ mice maintained on high fat diet for 10 weeks and harvested at 24 weeks of age and 16 week old chow fed mice was stained with Masson's Trichrome stain to visualize total plaque area. A, Stained sections spaced at 2-3 points through the aortic root were imaged and plaque area measured using image analysis software. B, Quantification of total plaque area within the aortic root from the chow fed animals showed no significant difference in total plaque area between hCD68GFP^{+} and ApoE$^{-/-}$ control animals. C, Analysis of Gal3 and mCD68 staining within the aortic root of the chow fed animals demonstrated no significant difference in macrophage content, as was seen in the high fat fed mice (Figure 1). Students T-Test, $p<0.05$ regarded significant, NS indicates no significant difference detected. However, a significant linear correlation between GFP^{+} area and macrophage staining as seen for both mCD68 and Gal3. n=5-8,

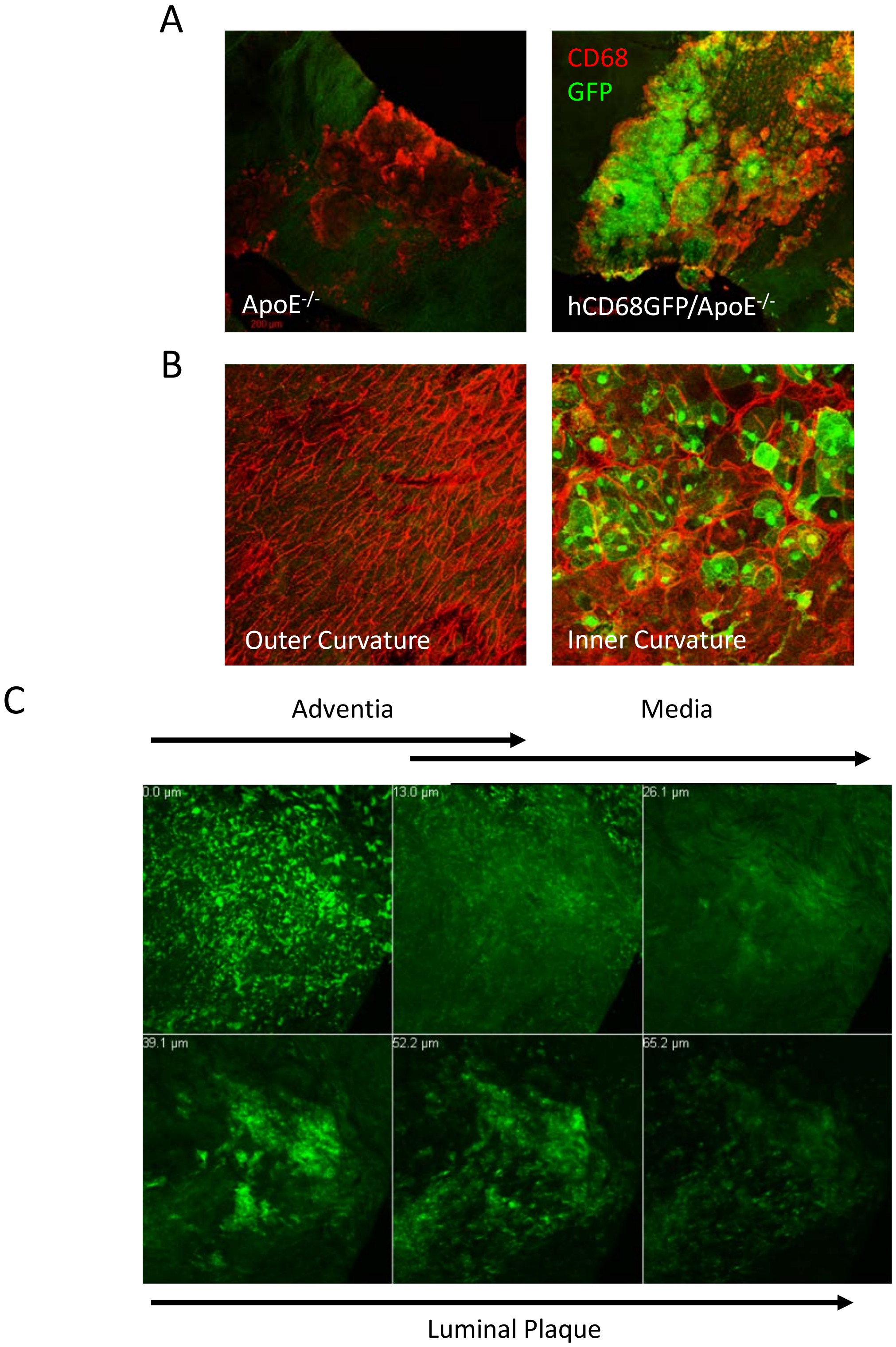

Supplementary Figure II : hCD-68GFP/ApoE$^{-/-}$ mice allow visualization of macrophages in aortic whole mount preparations. The aortic arch was perfused in situ with 4% paraformaldehyde, dissected clean of peri-vascular fat and post-fixed for 24 hours to prepare samples for en-face analysis by confocal microscopy. The arch was cut open along the outer curvature and notched to allow it to lie flat. A, the tissue was permeabilised with a blocking buffer and infiltrating macrophage were stained with ani-mCD68 (red). Autofluorescence of **the** elastic lamina was seen as a dim green signal in both tissues, but a very bright GFP$^+$ population of cell colocalising with the mCD68 signal (red) was seen in the hCD68GFP+ mice. B, Surface staining of endothelial cells (anti-CD31 – red) within en-face preparations from hCD68GFP/ApoE$^{-/-}$ mice showed the classical organized endothelial cell layer in non-disease prone areas of the outer curvature where no infiltration of GFP$^+$ cells was observed, whereas the atherosclerosis-prone inner curvature showed a disorganized endothelial cell layer associated with the infiltration of large GFP$^+$ myeloid foam cells (green). C, Confocal imaging was used to image the full wall depth and could detect both adventitial and luminal infiltration of hCD68GFP$^+$ macrophages in diseased areas, which could be clearly discriminated above the non-cell associated dim green signal associated with the elastic lamina in the vessel media.

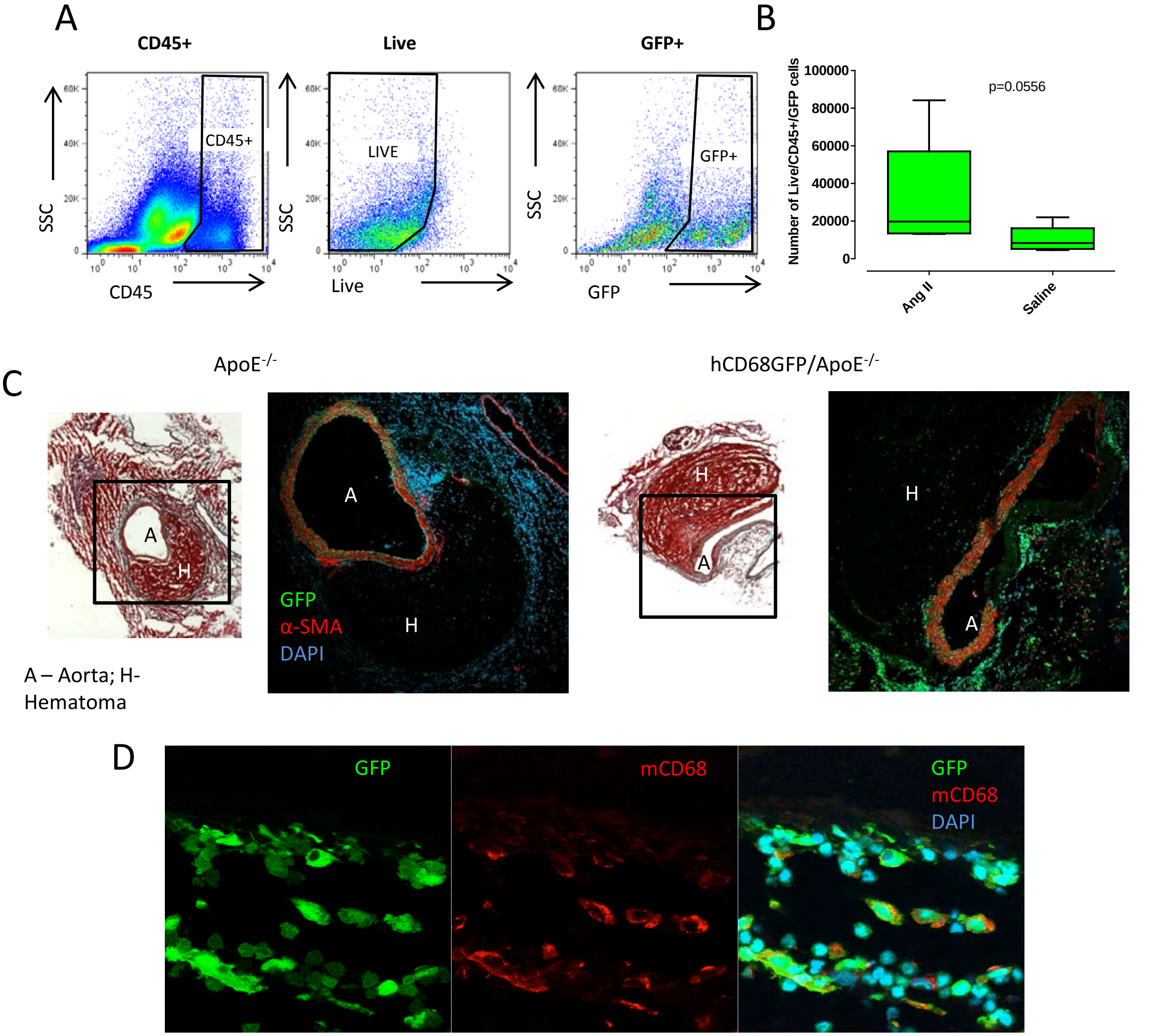

Supplementary Figure III : Infusion of Angiontensin II causes aortic inflammation and aneurysm formation. Aortic digests demonstrate the presence of GFP expression in leukocytes within the descending aorta from ApoE following Angiotensin II infusion (0.8mg/kg/day) for 5 days. Aortas were digested using a standard digest mixture (collagenase I, collagenase XI, hyaluronidase and DNAse I) with the resulting single cell suspension being stained with a viability dye and antibody cocktail to identify leukocytes (CD45) by comparison to isotype control samples or GFP- controls. A, viable leukocytes were identified as CD45$^+$/live cells and GFP+ cells were gated by comparison to GFP- samples. B, The number of Live/CD45/GFP$^+$ cells in Angiotensin II and saline treated mice (n=6 per group). A longer 14 day infusion of 0.8mg/kg/day angiotensin II resulted in the formation of aortic aneurysms. The aneurysmal tissue was excised and prepared frozen sectionning as described in the Materials and Methods. Masson Trichome (left panel) and immunofluorescence microscopy (boxed region shown in right panel) of serial sections demonstrates the presence of GFP$^+$ cells within the the vessel adventitia and perimeter of the hematoma within the aneurysm. The cellular area was identified by DAPI staining (blue) and the intact vessel wall using anti- α-smooth muscle actin antibody staining. Further sections were stained with anti-mCD68 to confirm the GFP$^+$ cells in the vessel adventitia demonstrated a strong co-localisation (D).

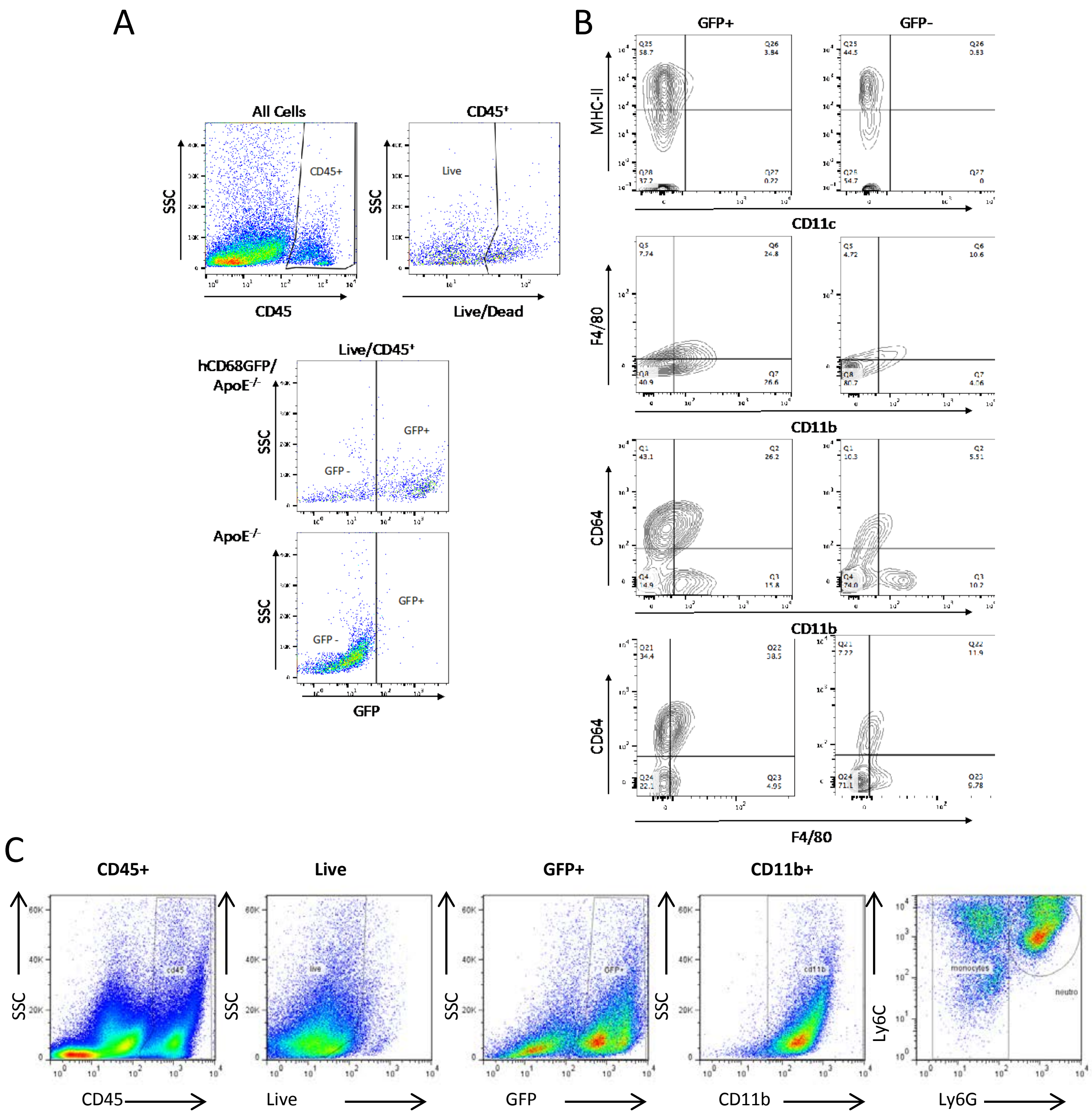

Supplementary Figure IV : hCD68GFP/ApoE$^{-/-}$ mice enable identification of multiple myeloid populations in aortic lesions in chow fed mice. Aortic Digests demonstrate the presence of GFP expression in multiple myeloid populations within the descending aorta from mice fed a chow diet and harvested at 16 weeks of age (female). Aortas were digested using a standard digest mixture (collagenase I, collagenase XI, hyaluronidase and DNAse I) with the resulting single cell suspension being stained with a viability dye and antibody cocktail to identify myeloid cell populations (CD45, CD11b, CD64, CD11c, MHC-II, F4/80) by comparison to isotype control samples or GFP- controls. A, Viable leukocytes were identified as CD45^{+}/live cells and GFP^{+} cells were gated by comparison to GFP- samples. B, Both GFP^{-} and GFP^{+} populations within the Live/CD45 population were gated to identify the presence of myeloid cell populations within these two populations. The MHC-II/CD11c, CD11b/F4/80 and CD64/CD11b populations were found primarily within the GFP^{+} population. Aortic digests demonstrate the presence of GFP expression in monocytes within the descending aorta following Angiotensin II infusion (0.8mg/kg/day) for 5 days. Aortas were digested as above and the resulting single cell suspension being stained with a viability dye and antibody cocktail to identify monocytes (CD45, CD11b, Ly6G, Ly6C) by comparison to isotype control samples or GFP^{-} controls. C, viable leukocytes were identified as CD45^{+}/live cells and GFP^{+} cells were gated by comparison to GFP^{-} samples. Monocytes were identified as Live/CD45^{+}/CD11b^{+}/Ly6G^{-}/Ly-6C^{+}

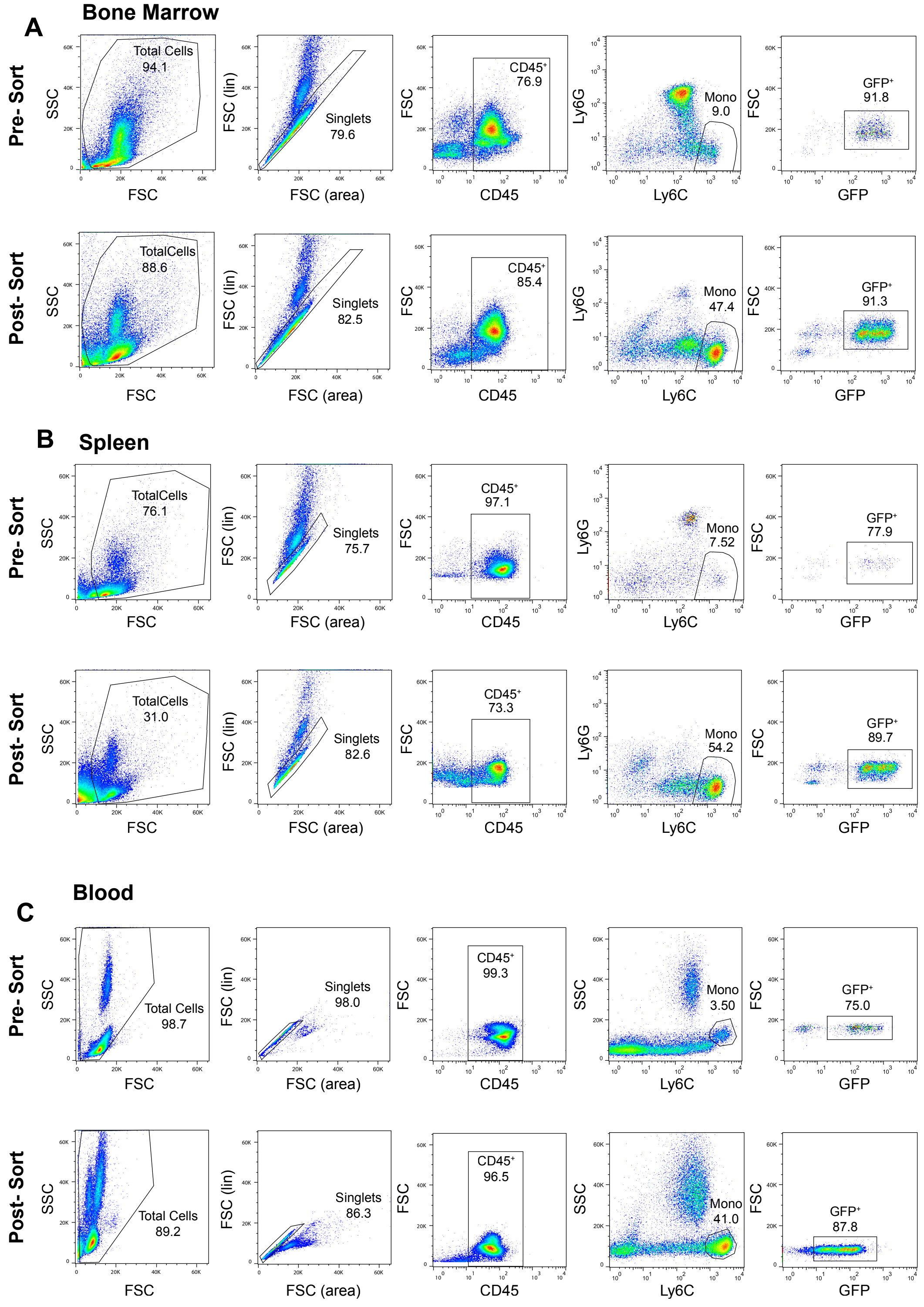

D

	Pre-Sort		Post-Sort		
	Total Count	*Total Monocytes*	*Total Count*	*Total Monocytes*	*% Yield Monocytes*
**Bone Marrow**	1.92×10^{8}	7.59×10^{6}	10.1×10^{6}	2.4×10^{6}	32
**Spleen**	1.86×10^{8}	1.36×10^{7}	5.0×10^{6}	2.0×10^{6}	15
**Blood**	1.52×10^{7}	5.11×10^{5}	8.8×10^{5}	2.7×10^{5}	52

Supplementary Figure V. Comparing monocyte yields and purity following isolation from blood, bone marrow and spleen. Monocytes were isolated from the (**A**) bone marrow; (**B**) spleen and (**C**) blood from 4 male hCD68GFP/ApoE$^{-/-}$ mice using negative immunomagnetic selection (see 'Methods'). Isolated monocytes were characterized as Ly6C^{high}/Ly6G^{low}, with a yield of 47% in bone marrow, of which 91% were positive for GFP. In the spleen a yield of 54% of isolated monocytes was obtained, of which 89% were positive for GFP. In the blood a yield of 41% of isolated monocytes was obtained, of which 87.8% were positive for GFP. (**D**) Total cell and total monocyte numbers are tabulated for post 'no touch' sorting from each tissue.

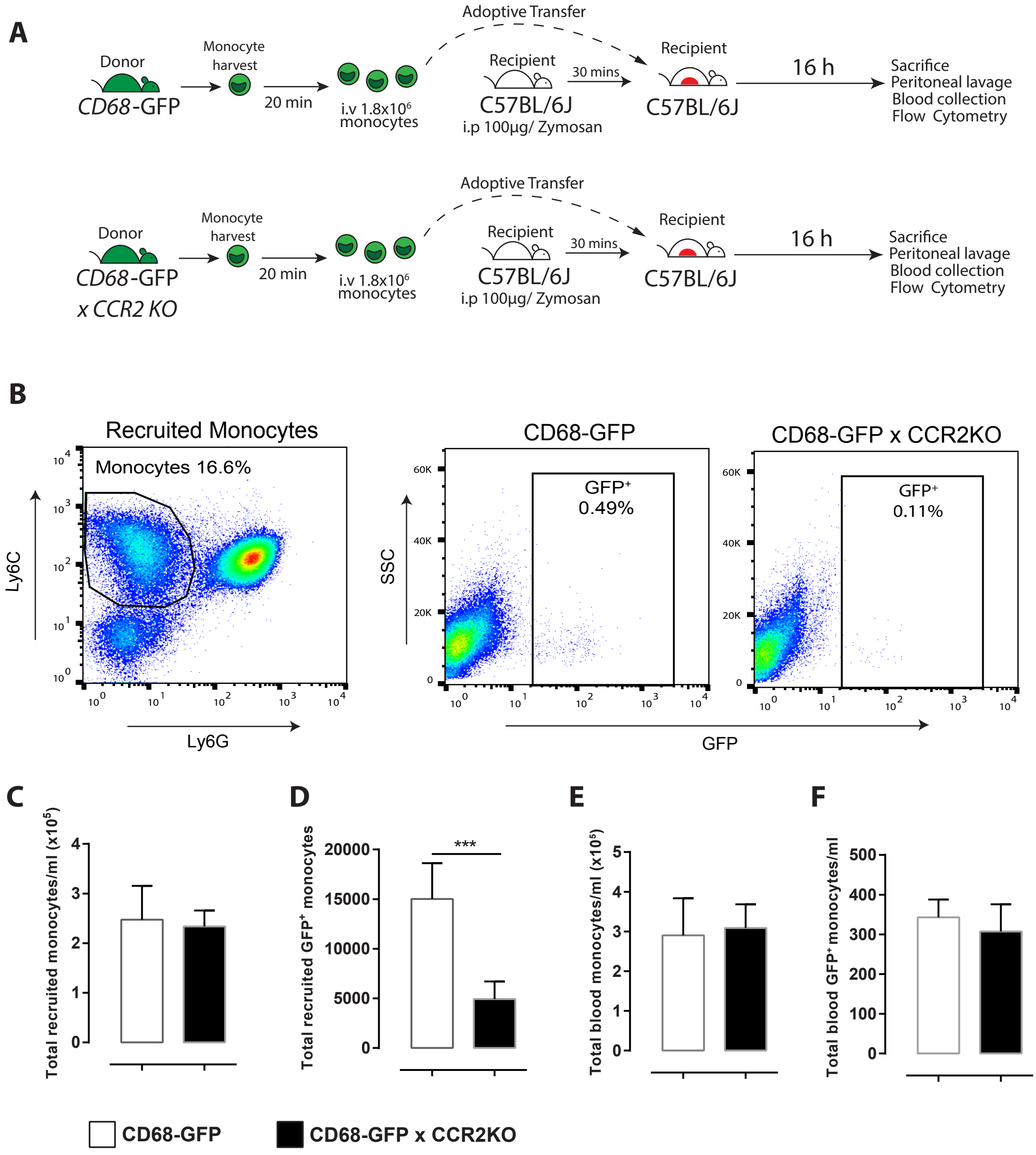

Supplementary Figure VI. Adoptive transfer of hCD68GFP/CCR2$^{-/-}$ monocytes and hCD68GFP monocytes (**A**) Diagram of the experimental design is provided. Monocytes were isolated from hCD68GFP and hCD68GFP/CCR2$^{-/-}$ using negative immunomagnetic selection. Isolated monocytes (1.8×10^6) were adoptively transferred into C57BL/6J mice by i.v. injection 30 min after i.p. injection with 100 µg zymosan. Mice were euthanized at 16 h, and peritoneal lavage and blood samples were collected. Statistical power calculations predicted that a group size of n=5 mice would be needed to detect a 20% biological effect size with a p value of <0.05 (**B**) Representative flow cytometry plots of peritoneal lavage from C57BL/6J mice that received adoptively transferred GFP positive monocytes from hCD68GFP or hCD68GFP/CCR2$^{-/-}$ during ongoing zymosan-induced peritonitis. Total monocytes and total adoptively transferred GFP$^+$ monocytes were quantified in peritoneal lavage (**C & D**) and blood (E & F). Data are expressed as mean + SEM of 5-6 mice from one independent experiment. Statistical analysis was conducted by paired Students T test. ***, p,0.001, relative to hCD68GFP/CCR2$^{-/-}$.

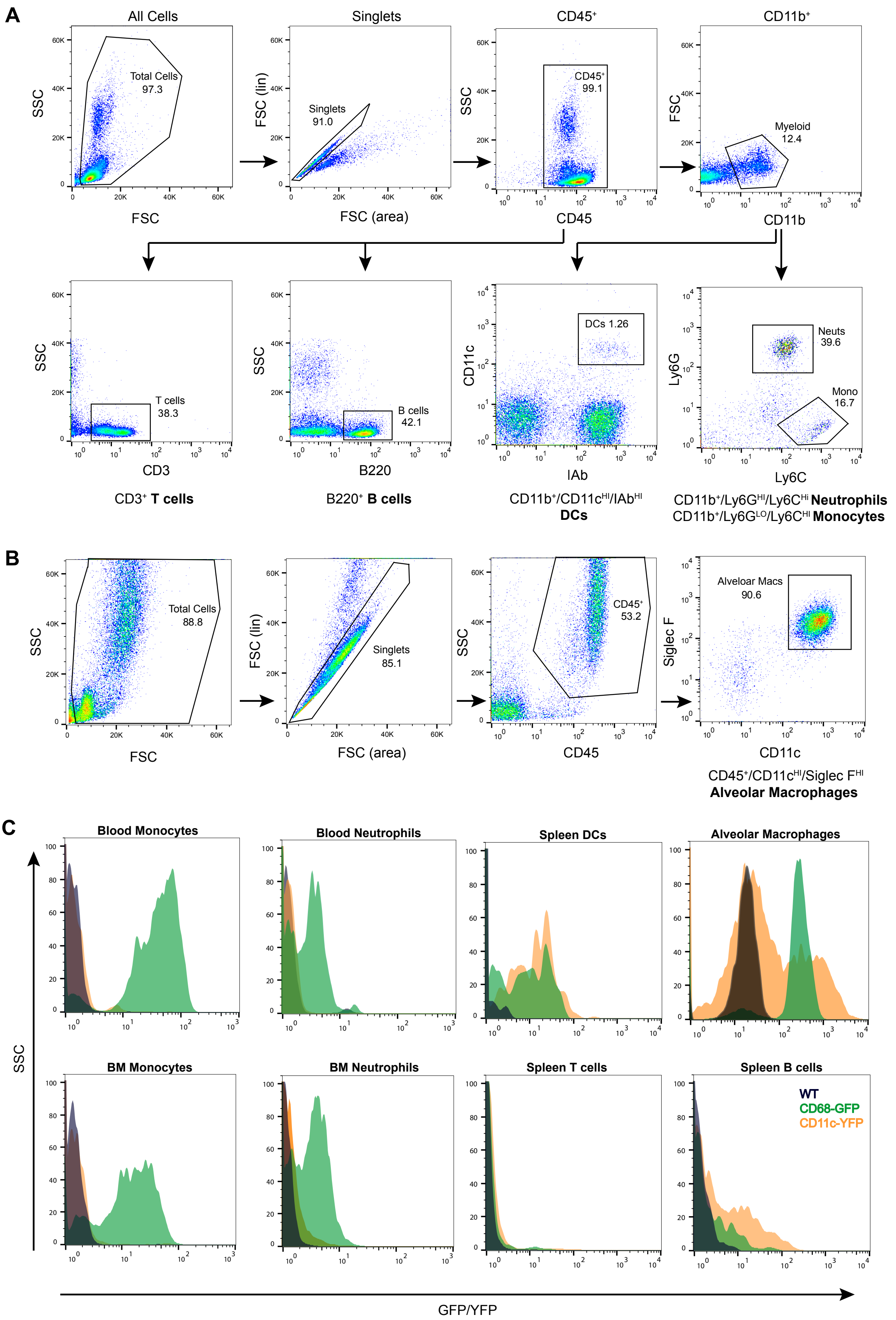

Supplementary Figure VII. Comparative flow cytometric analysis of GFP and YFP expression in hCD68GFP and CD11c-YFP reporter mice. (**A**) The staining and gating strategy used to characterize various leukocyte populations is shown and follows that used previously[5] (spleen was used to set the antibody panel). (**B**) Staining and gating strategy used to characterize alveolar macrophages from bronchoalveolar lavage (BAL). (**C**) hCD68GFP mice express GFP in blood monocytes, bone marrow monocytes and subset of blood and bone marrow neutrophils. In CD11c-YFP mice YFP expression was absent in all monocytes but present in splenic DCs, splenic B cells and alveolar macrophages in good agreement with previous reports[6]. WT mice were used as negative controls for GFP and YFP expression. Representative flow cytometry plots are shown; analysis was carried out in 2-3 mice per genotype.

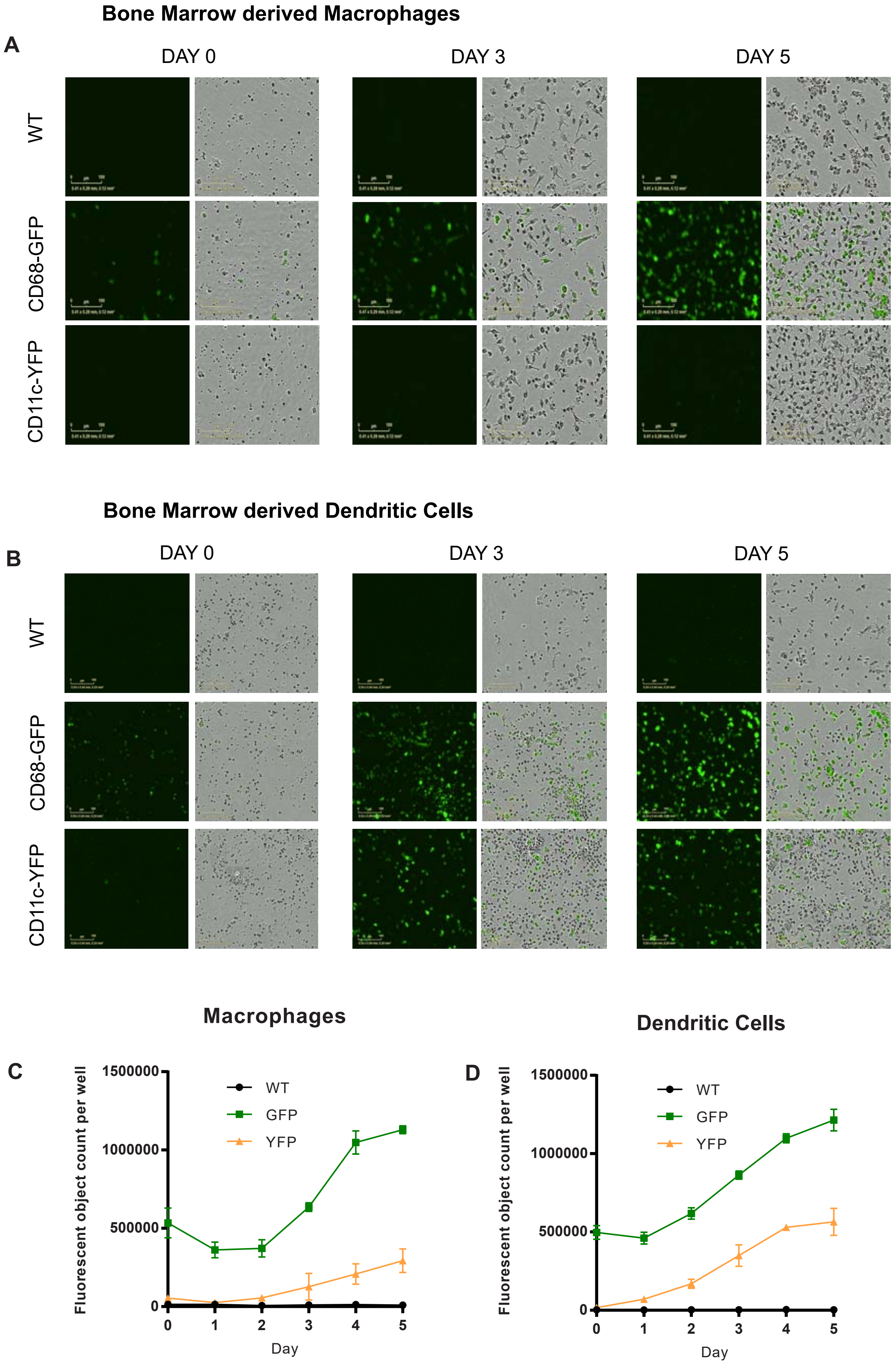

Supplementary Figure VIII. GFP and YFP transgene expression during monocyte to macrophage differentiation. Bone marrow cells ($2{\times}10^6$ total) from C57Bl6 (WT), hCD68GFP and CD11c-YFP mice were cultured *in vitro* in either MCSF conditioned media for macrophages or GM-CSF for DCs (DC culture was used as a positive control for the CD11c-YFP mice as YFP expression has been previously reported to increase following DC differentiation) Add citation here (**A & B**) Images were taken with the Incucyte Zoom platform every 8 hours a 5 day period. Representative phase contrast and green fluorescence are shown from Day 0, 3 and 5 of differentiation. GFP expression was maintained and increased in hCD68GFP monocytes differentiating to macrophages and DCs. In contrast, no YFP expression was observed in bone marrow monocytes isolated from CD11c-YFP reporter mice with an increase in transgene expression only observed during monocyte-to-DC differentiation. GFP and YFP expression in (**C**) macrophages and (**D**) DCs was also quantified using the florescent count object metric in the Incucyte Zoom software package. Data are expressed as mean ± SD, n=2 biological replicates with three technical replicates per genotype.
