# Supplementary material for: Tracking Monocyte Recruitment and Macrophage Accumulation in Atherosclerotic Plaque Progression Using a Novel hCD68GFP/ApoE−/− Reporter Mouse—Brief Report
Source: Arterioscler Thromb Vasc Biol. 2017 Jan 25;37(2):258–63. doi: 10.1161/ATVBAHA.116.308367 (PMC5274540; doi:10.1161/ATVBAHA.116.308367)
Supplement: Supplementary file 2 [file atv-37-258-s002.pdf]

Methods

hCD68GFP/ApoE$^{-/-}$ mice

hCD68GFP mice on a C57bl6/J background, overexpressing GFP under the control of the human CD68 promoter and IVS sequence[1], were crossed with C57bl6/J ApoE$^{-/-}$ mice [2] (Jackson laboratories, USA) to generate matched litters of hCD68GFP/ApoE$^{-/-}$ and ApoE$^{-/-}$ mice. Animals were housed in individually ventilated cages with 12 h light/dark cycle and controlled temperature (20–22°C). Standard chow (Harlan Ltd, UK), or a Western type high-fat diet (21% milk fat, 0.15% cholesterol, SDS Diets, UK) and water were available ab libitum. Genotyping of experimental mice was performed by standard PCR techniques to confirm the presence or absence of the ApoE gene and hCD68GFP transgene. Tissue was isolated from mice after culling with an overdose of anaesthetic (isoflurane >20%) and confirmation of death by either cervical dislocation or exsanguination. All animal procedures were approved and carried out in accordance with the University of Oxford Ethics Committee and the UK Home Office Animals (Scientific Procedures) Act 1986.

hCD68GFP/CCR2$^{-/-}$ and CD11cYFP mice

hCD68GFP mice were crossed with CCR2$^{-/-}$ mice to generate matched litters of hCD68GFP/CCR2$^{-/-}$ and CCR2$^{-/-}$ mice [3]. CCR2$^{-/-}$ and CD11cYFP mice were kept under the conditions described above[4]. All mice were on a C57bl6/J background.

Elicitation of peritoneal foam cells

Mice that had been maintained on a high fat diet for at least 8 weeks were injected ip with 1ml 2% Thioglycollate solution, in a modification of the published protocol [5]. 4 days following injection mice were culled and the peritoneal cavity lavaged with 10ml PBS containing 5mM EDTA. The resulting cells were transferred to Optimem media (Invitrogen, uk) (containing 0.2%BSA (low endotoxin, Sigma, UK) and plated onto uncoated glass coverslips. Cells were adhered for 2 hours, prior to the removal of non-adhered cells by washing with media. The remaining cells were fixed with 4% paraformaldehyde for 15 minutes then stained with Lipidtox red (Invitrogen) as per the manufacturer's insturctions, or with antibodies as below. Cells were counter stained with DAPI and imaged on a Zeiss LSM510 confocal microscope.

Histology and immunofluorescence microscopy of atherosclerotic lesions in frozen sections

Lesion size was assessed in OCT embedded frozen aortic root sections, which had been previously perfusion fixed with 4% paraformaldehyde, post-fixed overnight and dehydrated in 30% sucrose solution. The aortic root was sectioned at -20°C at 7μm per section. For sections stained with Masson-Goldner trichrome (Merck, Germany) the cut sections were post-fixed with Bouin's Fixative for 1 hour prior to staining according to the manufacturer's instructions. The average lesion size was calculated from sections taken at 80-100 μm intervals starting from the section showing all three aortic cusps. The infiltration of macrophages into aortic lesions was analysed using anti-mCD68 (Serotech, UK) or anti-Galectin 3 (Gal3 (Goat polyclonal AF1197, R&D Systems, UK) immunostaining, with Alexa 564 conjugated secondary antibodies (Invitrogen, UK). Plaque smooth muscle cells, endothelial cells and neutrophils were identified using α-smooth muscle actin (Clone 1A4 Sigma-Aldrich, UK), anti-CD31 (Clone MEC13.3 BD Biosciences, UK) or anti-S100a9 (Clone 2B10 Abcam) immunostaining. Positive staining was judged by comparison to isotype control antibodies used under the same conditions. Aortic lipid deposition was assessed in fixed aortas stained with Lipidtox Red (Invitrogen). The lesion area, Gal-3, CD68, and GFP positive areas were quantified from digitized microscopic images using Image-

Pro Plus. Co-localisation was quantified with ImageJ software and Manders co-efficient calculated for red and green channel pixel overlap.

En face whole mount aortic preparations

The aortic arch was perfused in situ with 4% paraformaldehyde, dissected clear of peri-vacular fat and post-fixed for 24 hours to prepare samples for en-face analysis by confocal microscopy. The arch was cut open along the outer curvature and notched to allow it to lie flat. For mCD68 staining the tissue was permeabilised with 0.1% Triton X-100 in a blocking buffer containing 1% gelatin and 10% goat serum, for cell surface CD31 staining the Triton X-100 was omitted. Tissues were preincubated in the blocking solution for 1-2 hours prior to incubation with anti-CD31 or mCD68 antibodies, followed by fluorescent secondary antibodies (Invitrogen, UK). Samples were mounted using Fluoromount G and imaged on a Zeiss LSM510 confocal microscope. Z-stack image series were compiled of the luminal side of the elastic lamina and stacked into a single image using the LSM Image Browser software. More detailed Z-series were taken across the full vessel wall using DAPI nuclei as a guide to wall morphology, and were used to create a movies of the transition of GFP+ cells from the adventitia to the luminal plaque, using the Zeiss Image browser.

Aortic Flow cytometry

Descending aortae were microdissected and digested in an enzyme solution containing 120 U/ml DNase I, 120 U/ml Hyalronidase, 750 U/ml Collagenase I and 250 U/ml Collagenase XI (all enzymes from Sigma-Aldrich, Gillingham, UK) at 37°C with constant mixing and intermittent disaggregation by pipette, until digestion had occurred (typically 30min – 45min) (modified from [6]). A single cell suspension was prepared by passing aortic pieces through a strainer prior to subsequent flow cytometry staining. Isolated aortic cells were stained with Fixable Live/Dead stain (Invitrogen, UK), followed by blockade of Fc receptors with a blocking antibody (2.4G2 BD Biosciences, UK). In the presence of the blocking antibody, cells were stained for surface markers including: CD45 (Clone 30-F11 BD Biosciences), anti-CD11b (Clone M1/70 BD Biosciences), CD64 (Clone X54-5/7.1 Biolegend), CD11c (N418 Biolegend), MHCII (1Ab subtype, Clone AF6-120.1 BD Biosciences), F4/80 (Clone FA11 Serotech, UK), Ly6C (Clone AL-21 BD Biosciences), Ly6G (Clone 1A8 BD Biosciences) with appropriate conjugated isotype controls. Blood samples were stained as above, prior to lysis and fixation in Whole Blood immunofluorescence solution (BD Biosciences, UK). Data was acquired using a CyAn Analyser flow cytometer (Beckman Coulter Ltd, UK) and then analyzed using FlowJo (Tree Star Inc, USA) software.

Isolation of primary mouse leukocytes for flow cytometry

Primary mouse bone marrow cells were isolated by flushing the tibiae and femurs with phosphate-buffered saline (PBS). The resulting cell suspension was gently disaggregated and passed through a 70 µm cell strainer to produce a single cell suspension. Spleens were disaggregated by passing them through a 70 µm cell strainer. Primary alveolar macrophages were recovered by bronchial alveolar lavage. Briefly, mice were killed and the trachea exposed. The thorax was opened to expose the lungs. A flexible plastic tube was inserted into the trachea then attached to a 2 ml syringe. The lungs were washed six times with 0.7 ml PBS/EDTA (10mM) by injection and withdrawal of solution. The success of the BAL procedure was judged by inflation of the lungs with the BAL solution on each of the washes. All primary cell suspensions and whole blood underwent red blood cell lysis (Pharmlyse, Invitrogen). Cells were resuspended in assay buffer (PBS supplemented with 2% FCS, 25 mM HEPES, 5 mM EDTA) and blocked with anti-mouse CD16/CD32 (0.5

µg/0.10^5 cells; Serotech) for 15 min on ice, stained with anti-mouse CD45 (2 µg/ml), anti-mouse Ly6C and anti-mouse Ly6G on ice protected from light for 30 mins. Cells were analysed on a CyAn™ ADP flow cytometer (Dako, Ely, U.K.).

Adoptive transfer of GFP monocytes
Monocytes were isolated from bone marrow using EasySep Mouse Monocyte Enrichment kit (Stem Cell Technologies). Briefly, femurs and tibias were harvested and flushed through with ice cold PBS. Bone marrow cell suspensions were passed through a 70-µm cell strainer to obtain a single cell suspension. Red blood cells were removed by hypotonic lysis (Pharmlyse, BD) according to the manufacturer's instructions. The bone marrow cell suspension was treated with the EasySep reagents and monocytes isolated by depletion using an EasyPlate magnet (Stem Cell Technologies). 1.5-1.8 × 10^6 isolated monocytes were injected in 100µl iv.

Monocyte isolation
Monocytes were isolated from bone marrow, spleen and blood using EasySep Mouse Monocyte Enrichment kit (Stem Cell Technologies). Briefly, femurs and tibias were harvested and flushed through with ice cold PBS. Bone marrow cell suspensions were passed through a 70-µm cell strainer to obtain a single cell suspension. Spleens were disaggregated by passing them through a 70 µm cell strainer and washed twice with PBS. Whole blood was collected by cardiac puncture into heparin (10I.U/ml) coated vacutainers. Red blood cells were removed by hypotonic lysis (Pharmlyse, BD) according to the manufacturer's instructions. The blood, bone marrow and spleen cell suspensions were treated with the EasySep reagents and monocytes isolated by depletion using an EasyPlate magnet (Stem Cell Technologies). Flow cytometric analysis was performed to assess purity and yield.

Bone marrow derived macrophages and dendritic cells
Bone marrow cells were isolated from femurs and tibias and a total of 1×10^6 cells were plated into 6 well plates and maintained in macrophage differentiation media (DMEM supplemented with 10% heat-inactivated fetal bovine serum (FBS), 2 mM l-glutamine, 100 U/ml penicillin and 15% supernatant from L929 cells as a source of macrophage colony stimulating factor) or in dendritic cell differentiation media (RPMI supplemented with 10% heat-inactivated fetal bovine serum (FBS), 2 mM l-glutamine, 100 U/ml penicillin, 1% sodium pyruvate and 5% supernatant from X63 cells as a source of granulocyte macrophage colony stimulating factor) for 5 days to stimulate differentiation. Cells were imaged every 8 hours over the 5 day period with the IncuCyte ZOOM imaging platform (ESSEN Bioscience, Germany).

Lipid and Lipoprotein analysis
Total Cholesterol and HDL cholesterol measurements were performed on heparinised plasma using cholesterol esterase and cholesterol oxidase/peroxidase enzymatic assays at Dept. Clinical Chemistry MRC Harwell (UK). Data are given in the test +/- SEM.

Ang II infusion by osmotic minipump
30-40 week old male hCD68GFP $ApoE^{-/-}$ mice were anaesthetized with isoflurane by inhalation and osmotic mini-pumps (Alzet Corp, USA) delivering saline or Ang II (Val5-Angiotensin II acetate salt hydrate, Sigma UK, 0.8 mg/kg/day) for 5 or 14 days were implanted subcutaneously. Aortic digests (5 day samples) or histology on aneurysmal tissue (14 day samples) were performed as above.

Statistical Analysis

Data are presented as mean ± SEM or box and whisker plots (max-min). Groups were compared using the Student's *t*-test for parametric data, assuming equal variance and normality of the data. When comparing multiple populations of aortic flow cytometry, data were analysed by analysis of variance (ANOVA) with the Dunn's Post-test for non-parametric data, as populations did not demonstrate equal variance. Linear regression was used to identify any significant association between GFP fluorescence and macrophage staining area, the r^2 statistic was used to indicate the 'goodness of fit' of the calculated relationship. A value of $P< 0.05$ was considered statistically significant.

References:

1. Iqbal AJ, McNeill E, Kapellos TS, Regan-Komito D, Norman S, Burd S, Smart N, Machemer DE, Stylianou E, McShane H, Channon KM, Chawla A, Greaves DR. Human cd68 promoter gfp transgenic mice allow analysis of monocyte to macrophage differentiation in vivo. *Blood*. 2014;124:e33-44
2. Piedrahita JA, Zhang SH, Hagaman JR, Oliver PM, Maeda N. Generation of mice carrying a mutant apolipoprotein e gene inactivated by gene targeting in embryonic stem cells. *Proc Natl Acad Sci U S A*. 1992;89:4471-4475.
3. Boring L, Gosling J, Cleary M, Charo IF. Decreased lesion formation in ccr2-/- mice reveals a role for chemokines in the initiation of atherosclerosis. *Nature*. 1998;394:894-897.
4. Lindquist RL, Shakhar G, Dudziak D, Wardemann H, Eisenreich T, Dustin ML, Nussenzweig MC. Visualizing dendritic cell networks in vivo. *Nat Immunol*. 2004;5:1243-1250
5. Li AC, Binder CJ, Gutierrez A, Brown KK, Plotkin CR, Pattison JW, Valledor AF, Davis RA, Willson TM, Witztum JL, Palinski W, Glass CK. Differential inhibition of macrophage foam-cell formation and atherosclerosis in mice by pparalpha, beta/delta, and gamma. *J Clin Invest*. 2004;114:1564-1576
6. Galkina E, Kadl A, Sanders J, Varughese D, Sarembock IJ, Ley K. Lymphocyte recruitment into the aortic wall before and during development of atherosclerosis is partially l-selectin dependent. *J Exp Med*. 2006;203:1273-1282
