# Supplementary material for: Tracking Monocyte Recruitment and Macrophage Accumulation in Atherosclerotic Plaque Progression Using a Novel hCD68GFP/ApoE−/− Reporter Mouse—Brief Report
Source: Arterioscler Thromb Vasc Biol. 2017 Jan 25;37(2):258–63. doi: 10.1161/ATVBAHA.116.308367 (PMC5274540; doi:10.1161/ATVBAHA.116.308367)
Supplement: Supplementary file 4 [file atv-37-258-s004.pdf]

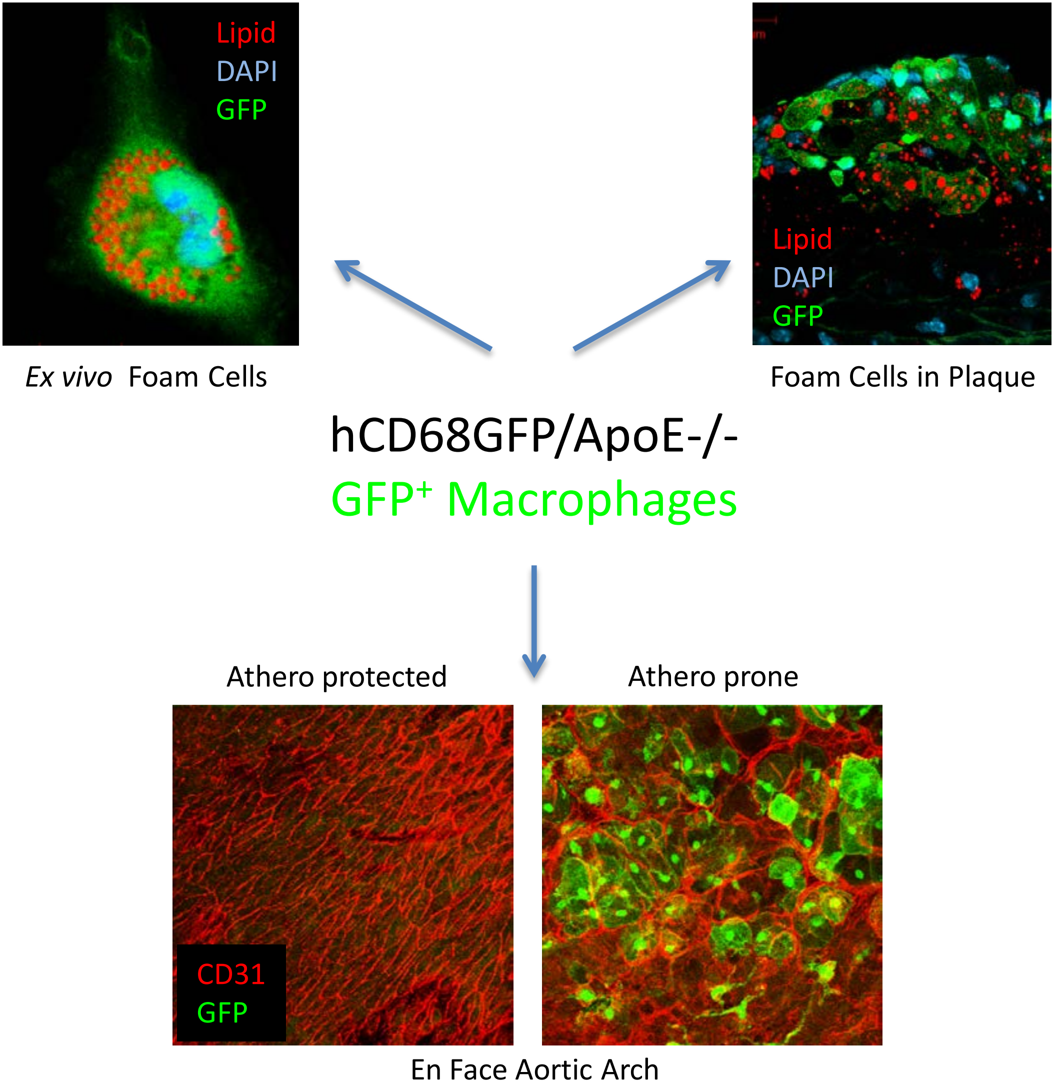
Lipid
DAPI
GFP
Ex vivo Foam Cells
Lipid
DAPI
GFP
Foam Cells in Plaque
hCD68GFP/ApoE-/-
GFP+ Macrophages
Athero protected
Athero prone
CD31
GFP
En Face Aortic Arch
